# The Rationale for Vitamin, Mineral, and Cofactor Treatment in the Precision Medical Care of Autism Spectrum Disorder

**DOI:** 10.3390/jpm13020252

**Published:** 2023-01-29

**Authors:** Neluwa-Liyanage R. Indika, Richard E. Frye, Daniel A. Rossignol, Susan C. Owens, Udara D. Senarathne, Andreas M. Grabrucker, Rasika Perera, Marielle P. K. J. Engelen, Nicolaas E. P. Deutz

**Affiliations:** 1 Department of Biochemistry, Faculty of Medical Sciences, University of Sri Jayewardenepura, Nugegoda 10250, Sri Lanka; 2 Autism Discovery and Research Foundation, Phoenix, AZ 85050, USA; 3 Rossignol Medical Center, Phoenix, AZ 85050, USA; 4 Rossignol Medical Center, Aliso Viejo, CA 92656, USA; 5 Autism Oxalate Project at the Autism Research Institute, San Diego, CA 92116, USA; 6 Department of Biological Sciences, University of Limerick, V94 T9PX Limerick, Ireland; 7 Bernal Institute, University of Limerick, V94 T9PX Limerick, Ireland; 8 Health Research Institute (HRI), University of Limerick, V94 T9PX Limerick, Ireland; 9 Center for Translational Research in Aging & Longevity, Texas A&M University, College Station, TX 77843, USA

**Keywords:** autism, vitamins, minerals, cofactors, micronutrients, supplements, nutrition

## Abstract

Children with autism spectrum disorder may exhibit nutritional deficiencies due to reduced intake, genetic variants, autoantibodies interfering with vitamin transport, and the accumulation of toxic compounds that consume vitamins. Importantly, vitamins and metal ions are essential for several metabolic pathways and for neurotransmitter functioning. The therapeutic benefits of supplementing vitamins, minerals (Zinc, Magnesium, Molybdenum, and Selenium), and other cofactors (coenzyme Q10, alpha-lipoic acid, and tetrahydrobiopterin) are mediated through their cofactor as well as non-cofactor functions. Interestingly, some vitamins can be safely administered at levels far above the dose typically used to correct the deficiency and exert effects beyond their functional role as enzyme cofactors. Moreover, the interrelationships between these nutrients can be leveraged to obtain synergistic effects using combinations. The present review discusses the current evidence for using vitamins, minerals, and cofactors in autism spectrum disorder, the rationale behind their use, and the prospects for future use.

## 1. Introduction

The revised version of the Diagnostic and Statistical Manual of Mental Disorders-5 (DSM-5) defines autism spectrum disorders (ASD) as a neuro-developmental disorder characterized by reduced social interaction and communication and restricted and/or repetitive patterns of behaviors or interests. ASD is known as a “spectrum” disorder because there is wide variation in the severity and the pattern of symptoms, progression, and prognosis.

ASD is accompanied by varying degrees of cognitive and language impairments and is associated with a number of medical conditions, genetic disorders, and other mental disorders [1]. The estimated global prevalence of ASD is 0.6% (95% confidence interval: 0.4–1%). American and European countries have a higher prevalence compared to Asian (0.4%) countries [2]. According to the biannual estimates reported by ASD and Developmental Disabilities Monitoring Network (ADDM), the prevalence of ASD in 8-year-old children in the United States has increased more than twofold over the decade from 2008 (11.3/1000) to 2018 (23/1000). ASD may persist into adulthood and require ongoing support. The etiological factors implicated in ASD pathogenesis are intricate [3]. For instance, a recent large exome sequencing study reported more than 100 autism-risk genes at a false discovery rate of 0.1 or less [4]. On the other hand, exposure to air pollutants, heavy metals, and pesticides has received attention as an environmental risk factor. The gene-environment interactions are also a focal point of study [5,6].

The etiology of ASD is complex, and treatments are limited. If identified early, (non)pharmacological interventions may improve core symptoms, co-occurring symptoms, and the ability to function [7]. Yet many behavioral and educational treatments have limited effectiveness, potentially due to underlying medical conditions which sometimes go undiagnosed. Some of these conditions include inadequate vitamin and mineral intake as well as metabolic abnormalities that can respond to vitamin and mineral supplementation. As such, at times, simple non-pharmacological interventions can be utilized to improve physiological health, which, in turn, can enhance the effectiveness of behavioral and educational interventions. Thus, this review appraises the evidence for nutritional interventions for children with ASD.

Children with ASD often have inadequate intake [8], lower blood levels of micronutrients [9], vitamins [10], and trace elements [11], higher rates of micronutrient deficiencies [12], reduced levels of vitamins in the brain [13], and antibodies against vitamin transporters at the blood-brain barrier [14]. Furthermore, ASD is associated with inflammatory bowel disease [15] and gastrointestinal (GI) inflammation increasing the risk for various nutritional deficiencies [16]. Scientific evidence also suggests that prenatal vitamin/multivitamin use and adequate intake of folic acid and Vitamin D are each associated with a lower likelihood of having a child with ASD [17], highlighting the fact that micronutrients play a vital role in modulating the risk of ASD during the early childhood as well as in intrauterine life.

The nutritional interventions that target metabolism in ASD include therapeutic diets and dietary supplementation of vitamins, minerals, polyunsaturated fatty acids, and probiotics [18]. A Canadian survey reported that seventy-five percent of children with ASD consumed nutritional supplements. Multivitamins (77.8%), vitamin D (44.9%), omega-3 fatty acids (42.5%), probiotics (36.5%), and magnesium (28.1%) were the most prevalent supplements. Other reported supplements included methylfolate, alpha-lipoic acid, sodium butyrate, N-acetyl cysteine, fluoride, adrenal cortex extract, selenium, milk thistle, liposomal curcumin, cannabidiol, and melatonin. The intention of supplementing with the above micronutrients was to enhance diet, promote immune system function, increase quality or duration of sleep, improve cognitive ability, decrease repetitive or restrictive behavior, promote sociability, increase interactions with others, and enhance motor skills. The factors against the supplementation were inadequate knowledge/information, cost, being considered harmful, etc. [19]. A study carried out by the Autism Speaks Autism Treatment Network revealed that multivitamin/mineral supplements were used by 56% of children with ASD. However, supplements did not correct the most common micronutrient deficits (vitamin D, calcium, potassium, pantothenic acid, and choline) [20]. This highlights the need for careful attention to micronutrient intake through a personalized approach.

Some supplements have been studied in controlled trials as monotherapy, while few studies used multi-nutrient supplements. For instance, Adams et al. studied the efficacy of a multi-micronutrient formulation that comprised several vitamins, minerals, choline, inositol, coenzyme Q_10_ (CoQ_10_), and N-acetyl cysteine [21], while another pilot study explored the effects of a combination of carnitine, CoQ_10_, and alpha-lipoic Acid (“mitococktail”) on mitochondrial function and neuro-behavioral performance in children with ASD [22]. However, one of the main limitations of these studies is not identifying the specific metabolic phenotypes that respond to nutritional supplementation. In addition, pathogenic mechanisms in ASD are vastly heterogeneous. Hence “one-size-fits-all” solutions are unlikely to benefit most children with ASD. Therefore, personalized approaches such as precision nutritional care are crucial. The following sections discuss the rationale behind vitamin, mineral, and cofactor therapies in ASD.

Vitamins are conventionally categorized as either water-soluble (B vitamins and Vitamin C) or fat-soluble (Vitamin A, D, E, and K). Water-soluble vitamins are generally believed to have low toxicity; hence, doses higher than recommended can usually be safely delivered [16]. Higher doses can be given in suspected or proven clinical deficiency, for patients at risk of deficiency, and in patients with deficiencies of other group-B vitamins [16].

Active forms of many vitamins and some trace elements (iron, magnesium, copper, zinc, selenium, and molybdenum) are essential cofactors for numerous enzymes involved in metabolism, antioxidant defense, and redox activity. In addition, non-vitamin coenzymes such as CoQ_10_, alpha-lipoic acid, and tetrahydrobiopterin are synthesized in the body but are also available in the diet or as nutritional supplements.

## 2. Vitamins

### 2.1. Vitamin B1

The activated form of Vitamin B1 (thiamine) is called thiamine pyrophosphate (TPP), which is a cofactor for several enzyme complexes in energy metabolism, including pyruvate dehydrogenase (PDH), alpha-ketoglutarate dehydrogenase and branched-chain alpha-keto acid dehydrogenase (BCKDH) complexes. The enzyme transketolase, which links the pentose phosphate pathway with the glycolytic pathway, also uses TPP. Furthermore, the thiamine-dependent peroxisomal enzyme, 2-hydroxy acyl-CoA ligase, is required for the metabolism of 3-methyl branched fatty acids and 2-hydroxy straight-chain fatty acids [23]. Recently, scientists have discovered that thiamine has extensive non-cofactor functions. Thiamine and/or its derivatives are allosteric regulators of malate dehydrogenase, glutamate dehydrogenase, and pyridoxal kinase (PDXK). Therefore, thiamine is not only a coenzyme for acetyl-CoA synthesis but also an allosteric regulator of its synthesis. Thiamine and its derivatives are also involved in the regulatory acetylation of proteins, acetylcholine biosynthesis, receptor activity, cholinergic neurotransmission, regulation of metabolic enzymes, antioxidant effects, and neurotrophic effects on the brain [24,25].

Four major conditions of thiamine depletion: Wet Beriberi, dry beriberi, Wernicke’s encephalopathy, and Korsakoff syndrome, can result in severe disease or even be fatal. The deficiency mainly affects the brain due to its dependence on glucose oxidation for energy and the slow thiamine transport across the blood-brain barrier [23,26]. Thiamine absorption can also be impaired in folate deficiency [16].

A single study reported that 11% of ASD subjects without any vitamin supplements had whole blood thiamine levels below the reference intervals, while 20% had levels above normal reference intervals [27]. Another study revealed that thiamine and thiamine monophosphate concentrations are not significantly different from healthy subjects, while TPP levels were decreased by 24% in ASD children, suggesting the decreased release of TPP from cells and/or impaired uptake of TPP from the GI tract [28]. Furthermore, thiamine deficiency associated with Wernicke’s encephalopathy and dysautonomia has been reported in several cases of ASD [29,30]. These dysfunctions may appear less severe in mild to moderate ASD.

Children with ASD have a higher risk of obesity [31,32]. On the other hand, obesity is associated with thiamine deficiency [33]. A Thai study revealed a 42% prevalence of thiamine deficiency among obese Thai children. Most of those cases were subclinical [34]. An imbalance between the overconsumption of carbohydrates, and the availability of thiamine, may induce a type of relative thiamine deficiency, also called high-calorie malnutrition [35]. Lonsdale highlights that thiamine deficiency can have profound consequences in the brain due to impaired phytanic acid and long-chain fatty acid metabolism and the downstream effect on beta-oxidation [36]. Correspondingly, dysautonomia associated with thiamine deficiency in two siblings with ASD reoccurred after ingestion of sugar, milk, or wheat [30]. Self-imposed dietary restrictions may contribute to the inadequate thiamine intake reported in ASD individuals [9,29].

Furthermore, individuals with ASD may exhibit increased sulfite levels due to impaired sulfite oxidase activity [37]. Interestingly, sulfite is well-known to consume thiamine [38]. Taken together, individuals with ASD may benefit from thiamine supplementation, given that ASD can be associated with thiamine deficiency, self-imposed dietary restrictions, obesity, and increased sulfite levels.

In a pilot study, after two months of thiamine tetrahydrofurfuryl disulfide (TTFD) supplementation, eight out of the ten children with ASD clinically improved as measured by the Autism Treatment Evaluation Checklist (ATEC) [30]. However, more randomized clinical trials are needed to corroborate the use of thiamine in ASD.

The most ordinary and available form is thiamine hydrochloride. It is generally the cheapest but may have less potency, with the available evidence being outdated. Commonly, it is the form used in a study if the form is not specified. TTFD is a synthetic version of thiamine found in garlic called allicin. Its disulfide bond allows it to cross a membrane without the help of a transporter. Benfotiamine is another newer popular form of thiamine that also has advantages in crossing cell membranes. Derrick Lonsdale has considered TTFD the preferred form for his research on thiamine in ASD [30].

The estimated adequate requirements (EAR) of thiamine in children vary between 0.7 and 1.2 mg/day, while the recommended dietary allowance (RDA) is 0.9 to 1.2 mg/day. Thiamine is well absorbed and can be administered by oral, enteral, or intravenous route [16]. A 50–100 mg daily dose has been commonly used in ASD [39].

Current practices have failed to identify that therapeutic thiamine supplementation can be life-changing. On the other hand, higher doses need to be tried because thiamine is now known to have extensive roles beyond its cofactor functions. Unfortunately, since this is a newer strategy, the long-term effects of such high doses are yet to be evaluated. Thiamine should be considered whenever there are concerns with energy homeostasis, mitochondrial function, or slow onset of neurological defects in ASD. While there is no evidence of toxic levels of thiamine reported in humans, there is extensive literature on the significant or even permanent damage that may occur in the absence of adequate thiamine. However, allergic reactions (anaphylactic shock) have been reported as a rare complication of intravenous thiamine administration [23].

### 2.2. Vitamin B2

Vitamin B2 (Riboflavin) is essential for the formation of both flavin mononucleotide (FMN) and flavin adenine dinucleotide (FAD), which account for most of the riboflavin in plasma and tissues. In addition, riboflavin is involved in redox reactions, antioxidant functions, antibody production, immunomodulation, the electron transport chain, fatty acid beta-oxidation, choline catabolism, purine catabolism, myelin synthesis and metabolism of other B vitamins such as B3, B6, B9 and B12 [16,40]. The enzymes that depend on riboflavin as a cofactor to function which are particularly relevant in ASD include xanthine oxidase, succinate dehydrogenase, glutathione reductase, methylene-tetrahydrofolate reductase (MTHFR), and pyridoxine phosphate oxidase. Considering the fundamental role of riboflavin in reducing oxidative stress, mitochondrial function, and myelin formation, this vitamin could be regarded as a putative protective agent in ASD. Despite all that interest, vitamin B2 as a monotherapy in ASD has not been studied in a controlled trial. In ASD, riboflavin may be given at 100 to 400 mg daily, as described in the literature.

### 2.3. Vitamin B3

Nicotinamide is the precursor for nicotinamide adenine dinucleotide (NAD+) and nicotinamide adenine dinucleotide phosphate (NADP+), the coenzymes essential for numerous metabolic reactions in the body, notably in energy metabolism [41]. Niacin, also known as nicotinic acid, is converted to niacinamide/nicotinamide in the body [42].

In a single study, mean blood vitamin B3 levels in individuals with ASD were not significantly different from that of neurotypical controls. However, there was a significant number of ASD individuals with values above and below the reference intervals suggesting the presence of multiple endophenotypes [27]. Individuals with ASD are vulnerable to developing vitamin deficiencies, including vitamin B3 deficiency, due to selective eating practices [43]. Conversely, individuals with ASD with a defective tryptophan-serotonin-melatonin pathway, such as those with reduced acetylserotonin methyltransferase (ASMT) activity, may have an accelerated tryptophan-nicotinamide pathway [44].

In a urine metabolomic study, nicotinamide and metabolites in the tryptophan-kynurenine pathway were identified as biomarkers that discriminate between ASD patients and controls [45]. Urinary excretion of N-methyl-2-pyridone-5-carboxamide, N-methyl nicotinic acid, and N-methyl nicotinamide was increased in individuals with ASD, indicating increased nicotinic acid degradation [44]. Since whole blood niacin levels were remarkably similar in the ASD and neurotypical groups, and whole blood niacin levels did not significantly correlate with N-methyl nicotinamide levels, N-methyl nicotinamide is suggested as a more sensitive assay [27].

Interestingly, the degradation of nicotinamide involves methylation reactions that require S-adenosylmethionine (SAMe) as the methyl donor to produce N-methyl-nicotinamide and N-methyl-2-pyridone-5-carboxamide [46]. The endophenotype with increased N-methyl nicotinamide excretion may be at risk of SAMe depletion or reduced methylation capacity, a consistent metabolic abnormality reported in ASD [47].

A daily dose of 50–100 mg of vitamin B3 has been commonly used to treat mitochondrial dysfunction in ASD [39]. Nicotinamide has fewer undesirable side effects compared to its biochemical precursor, niacin, such as skin flushing, pruritis, and xerosis [42].

### 2.4. Vitamin B5

Vitamin B5 (Pantothenic acid) is required to synthesize coenzyme A (CoA), a thiol that reacts with carboxylic acids to form thioesters such as acetyl-CoA and succinyl-CoA in the body [41]. Deficiency and abnormal metabolism of vitamin B5 are linked to ASD. For instance, *DIP2A* encoding disconnected-interacting protein homolog 2 A is an ASD candidate gene involved in acetyl-coenzyme A synthesis. The gene is primarily expressed in the brain regions with abundant pyramidal neurons. Deficiency of DIP2A may lead to impaired maturation of dendritic spines and defective synaptic transmission in an acetyl-coenzyme A-dependent manner [48].

Pantothenic acid, biotin, alpha-lipoic acid, and to a lesser degree iodine cross cell membranes using a sodium-dependent multivitamin transporter (SMVT/SLC5A6). This is the main mode of biotin and pantothenic acid uptake into brain capillary endothelial cells. Since this is a multivitamin transporter, vitamins can compete for transportation [49].

Several studies report that a high proportion of individuals with ASD have inadequate vitamin B5 intake [20,50,51]. Furthermore, urinary metabonomic studies reported lower levels of urinary vitamin B5 in children with ASD compared to their healthy controls [52,53]. Interestingly, children and adolescents with ASD traits have been reported to have lower intakes of vitamin B5 compared to individuals without ASD [54]. In line with the evidence mentioned above, the mean vitamin B5 blood levels have been reported to be significantly lower in individuals with ASD than in neurotypical individuals, while 11% of individuals with ASD had values below the reference range [27]. A randomized, double-blind, placebo-controlled (DBPC) three-month vitamin/mineral treatment trial conducted by the same research group revealed that vitamin B5 blood levels could be improved by supplementation [21].

Even though there is limited scientific evidence to support deficiency or abnormal vitamin B5 metabolism as a causative factor in ASD, this could be a possible contributory factor that could be targeted in therapeutics. Therefore, vitamin B5 is a good candidate for a multivitamin supplement or therapeutic cocktail for ASD. Furthermore, as pantothenic acid toxicity is rare, it can be given at 5–1200 mg in 1 to 2 doses per day.

### 2.5. Vitamin B6

Vitamin B6 is found in six forms: pyridoxal, pyridoxine, pyridoxamine, pyridoxic, and their respective phosphorylated forms synthesized by the action of PDXK. Pyridoxic acid is the catabolic product of vitamin B6. Phosphorylation not only forms the active cofactor, pyridoxal-5-phosphate (PLP) but also modulates B6 trafficking. PLP is a coenzyme required for carbohydrate, lipid, amino acid, and heme metabolism [16,55]. Beyond coenzyme functions, it is also a potent antioxidant, metal chelator, and carbonyl scavenger [56].

Many enzymes involved in synthesizing neurotransmitters, including GABA, serotonin, dopamine, noradrenaline, histamine, glycine, and d-serine, are vitamin B6-dependent [57], indicating that vitamin B6 supplementation may enhance many neurotransmitter systems in individuals with ASD [58]. For instance, Vitamin B6-dependant glutamate decarboxylase [59] irreversibly converts glutamate to gamma-aminobutyrate (GABA), a point of discussion in the pathogenesis of ASD. GABA has been targeted by pharmacological agents attempted in ASD [60].

Furthermore, cystathionine beta-synthase, cystathionine gamma-lyase in the transsulfuration pathway [57], and cysteine sulfinic acid decarboxylase in taurine biosynthesis [61] are B6-dependant enzymes. Abnormal transsulfuration metabolism and reduced antioxidant capacity remain critical areas for research in ASD [62].

Cysteine desulfurase is a B6-dependent enzyme critical for forming iron-sulfur centers like in the respiratory chain complexes I, II, and III and both mitochondrial and cytosolic aconitase [63]. Another B6-dependent desulfurase is molybdenum cofactor sulfurase (MOCOS) which was found to be under-expressed in some individuals with ASD due to an antisense long noncoding RNA [64].

B6 deficiency can disrupt the immune system by changing the balance between two types of T-helper cells (Th1 vs. Th2) towards an excessive Th2 response [65]. This Th1 to Th2 shift has for many years been considered a concern in ASD [66]. In addition, the immune system may also be affected as the important immune factor sphingosine-1-phosphate (S1P) is regulated by S1P lyase, a B6-dependent enzyme. When this lyase is deficient, lymphocytes are arrested, followed by lymphopenia, and immunosuppression, with the release of pro-inflammatory cytokines enhancing inflammatory processes [67].

Dephosphorylated forms of vitamin B6 are absorbed from the intestine into the blood by passive diffusion [16]. In the liver, PLP becomes tightly bound to serum albumin before being secreted into the circulatory blood system. In addition to PLP (about 70–90%), plasma also contains pyridoxal (8–30%). Humans have no vitamin B6-specific transporter. The phosphate group attached to vitamin B6 creates a barrier to its movement into or out of a cell. Tissue non-specific alkaline phosphatase (ALPL) outside the cell membrane removes the phosphate group from PLP, pyridoxine-5-phosphate, or pyridoxamine-5-phosphate, allowing any of them to cross into the cell [55]. Once inside, PDXK adds a phosphate group, allowing the molecule to stay inside the cell.

Even though individuals with ASD demonstrated improvements with B6 supplements [68], Adams et al. in 2006 found that children with ASD who were not supplemented with B6 had paradoxically high plasma levels of total vitamin B6 (phosphorylated and non-phosphorylated). It was proposed that this elevation came from a failure of the PDXK activity resulting in reduced conversion of pyridoxal and pyridoxine to phosphorylated vitamin B6 [69]. However, in a study of genetic PDXK deficiency which was not published until 2019, blood levels of PLP were found to be low in patients with biallelic mutations in the PDXK gene. All the other vitamers of B6 tested in these patients were normal or zero instead of raised [70]. These data are inconsistent with Adam’s hypothesis.

If inhibition of ALPL occurs in ASD, then we might expect either common ALPL gene defects or an ALPL inhibitor. Vanadate and other oxyanions are found to be ALPL inhibitors [71]. Is there another oxyanion consistently elevated in ASD? Indeed, in 2011, Konstantinowyc et al. found consistently higher levels of oxyanion oxalate in blood plasma in children with ASD; 3-fold greater than controls, and urinary oxalate concentrations were 2.5-fold higher in 24-h urine. Their results showed no increased risk for kidney stones using the Bonn Risk Index [72].

It has been known since 1976 that red blood cells transport pyridoxal-5-phosphate via the transporter called Band 3, produced by SLC4A1 [73]. Work by Lawrence Solomon confirmed that red blood cells contain apoenzymes that require B6. PLP was thought to enter the erythrocyte by binding hemoglobin, but this binding would keep it from being activated, and pyridoxine itself would not lead to the activation of AST [74]. Band 3 is also a sulfate and an oxalate transporter or exchanger, meaning that its substrates can be inhibitors of each other [75], which could indicate why the elevated oxalate in plasma in children with ASD might influence the transport of pyridoxal-5-phosphate in red blood cells. High levels of pyridoxal-5-phosphate in the blood may change the regulation of the other substrates of Band 3. Albumin levels may also modify this influence of pyridoxal phosphate concentration in plasma and route more pyridoxal phosphate to red blood cells [76]. Furthermore, a study in rats confirmed that B6 deficiency lowered both AST and ALT and, at the same time, almost doubled oxalate [77].

Few treatments have demonstrated as much effectiveness in ASD as B6 supplementation. One study reported finding 18 studies on efficacy and safety and 11 DBPC studies over 35 years [78], but B6 is also a vitamin with considerable concern about potential toxicity.

Pyridoxine is the standard form used in most supplements. Two studies described a paradox, showing that high concentrations of pyridoxine led to increased intracellular concentrations of pyridoxine phosphate, which competes with PLP for apoenzyme binding, decreased vitamin B6 function, and even cell death, suggesting that pyridoxamine and pyridoxal are more effective than pyridoxine in increasing residual enzyme activity [79,80]. Furthermore, another study looked at healthy controls using a single 200 mg oral dose of pyridoxamine and compared that to three staggered doses of pyridoxamine at three daily doses of 67 mg. Both schedules increased pyridoxal and PLP without elevating pyridoxine [81]. Though this may encourage us to think this form would be preferred, the FDA has been erecting barriers to using pyridoxamine as a supplement, wanting to make it instead a pharmaceutical, giving it limited availability and increased cost. Hadtstein et al. described pyridoxine toxicity as symmetric, progressive impairments in touch, pinprick, temperature, vibrational, and positional sense in the extremities [82]. If the vitamer is used and the dose is chosen wisely, a trial of B6 may yield what often has been reported as significant improvements in those with ASD.

Unfortunately, monitoring PLP or any other single B6 vitamer in plasma does not predict cellular levels, and the distribution and quantity of B6 vitamers were found to be different by cell type [55]. Even age, pregnancy, high protein diet, inflammatory conditions, and plasma glucose levels can affect the transformation and distribution of vitamin B6 forms and change the testing interpretation [83,84].

### 2.6. Vitamin B7

Vitamin B7 (Biotin) is critical for energy production and storage as it is a cofactor for several carboxylases involved in fatty acid synthesis, branched-chain amino acid catabolism, and gluconeogenesis [41]. For instance, acetyl-CoA carboxylase catalyzes the critical regulatory step of fatty acid synthesis. Propionyl-CoA carboxylase catalyzes the conversion of propionyl-CoA, a product of branched-chain amino acid catabolism, to methyl malonyl-CoA, which enters the citric acid cycle through conversion to succinyl-CoA [85]. Pyruvate carboxylase is an anaplerotic enzyme that converts pyruvate to oxaloacetate, an intermediate in the citric acid cycle and a precursor for gluconeogenesis [86].

A systematic review and bioinformatic analysis revealed that miRNAs in ASD target autism-risk genes involved in fatty acid metabolism, fatty acid biosynthesis, lysine degradation, biotin metabolism, and steroid biosynthesis [87]. Furthermore, biotinidase deficiency has been reported in subjects with ASD. Biotinidase is an enzyme needed to recycle biotin; hence these patients can be treated with biotin supplements [88,89]. Patients with partial biotinidase deficiency may present later with ASD symptoms without hair loss or skin manifestations [90,91]. A case report of two siblings highlights that diagnosis of partial biotinidase deficiency at the age of 4 and, consequently, delayed biotin supplementation was associated with a poor outcome. In contrast, early identification of the same disease at birth and early biotin supplementation prevented the younger sibling’s behavioral abnormalities and developmental delay [92]. Another 10-year-old child with ASD with poor nail and hair growth was treated successfully with biotin, 10 mg/day initially, and subsequently increased up to 25 mg/day. Following treatment, her social skills, academic performance, and artistic skills improved dramatically and enabled her to function normally within a mainstream academic and social school environment. However, her biochemical investigations revealed normal ammonia, amino acids, and organic acids, while enzymatic biotinidase testing was normal. Biotin levels were not carried out [93]. Additionally, an infant with ASD, biotinidase-deficient and drug-resistant epileptic encephalopathy improved dramatically following biotin supplementation [94]. Thus, mild to moderate biotin deficiencies may manifest in ASD with less severe neurological disabilities and without characteristic skin and manifestations of biotin deficiency.

Individuals with ASD with inherited metabolic defects in biotin-dependent enzymes, including those with propionyl-CoA carboxylase deficiency (propionic acidemia) and 3-methylchrotonyl-Co-A carboxylase deficiency, have been reported [95,96,97]. A patient with 3-methylchrotonyl-Co-A carboxylase deficiency treated with carnitine, biotin, and special nutritional therapy demonstrated improvements in hyperactivity and speech [97]. Oxalate, known to be elevated in a subgroup of ASD, is the most potent inhibitor of pyruvate carboxylase [98]. Pyruvate carboxylase plays a crucial role in the citric acid cycle, gluconeogenesis, lipogenesis, and biosynthesis of neurotransmitters [86]. In the aforementioned situations, biotin can potentially increase the residual enzyme activity as long as the biotin-binding domain is intact.

Interestingly, a study reported that CpG loci were hypomethylated in *PCCB*, the gene encoding propionyl-CoA Carboxylase subunit beta (PCCB) in individuals with ASD as compared to controls while the organic acid analysis of the ASD group with hypomethylated *PCCB* gene revealed elevated metabolites suggestive of mitochondrial dysfunction [99]. This could be linked to the reduced methylation capacity [47] and the hypomethylation of DNA [99] observed in ASD.

Magnesium biotinate, a novel biotin complex with superior absorption, has been effective in decreasing the expression of neurotoxicity-related cytokines, improving the brain and serum magnesium, biotin, serotonin, and dopamine concentrations, and ameliorating dysfunctions in social behavior, learning, and memory in propionic acid-exposed rats with ASD behaviors [100]. A study conducted in the USA reported that biotin levels of ASD were 20% lower compared to neurotypical controls, and nearly 7% of those with ASD had biotin levels below the reference range [27]. The same study group reported that supplementation with a formulation made of multiple minerals and vitamins resulted in a 51% increase in whole blood biotin levels [21]. In light of the multiple levels of evidence pointing towards treatable abnormalities related to biotin metabolism, more research should explore its potential role in improving core symptoms or associated symptoms of ASD.

A defect in the *SLC19A3* gene encoding a thiamine transporter is known to produce an often-fatal condition if it is not treated, called biotin-thiamine-responsive basal ganglia disease. This gene is responsive during stress and particularly depends on biotin [101]. Initially, this disorder was successfully treated with extremely high doses of biotin, but more recently, the disorder was recognized as a thiamine transport defect, leading to treatment centered on high-dose thiamine and the discovery that biotin was needed for the transporter to integrate into the cell membrane. Membrane localization is required for it to transport thiamine [102]. For this reason, acquired biotin deficiencies secondary to antibiotics use could impair the absorption of thiamine during critical periods of brain growth and maturation. Therefore, it is advisable to investigate and correct a possible concomitant biotin deficiency in a patient with thiamine deficient-ASD.

Biotin can be given orally or intravenously. Toxicity is unlikely [16]. Poor nail/hair growth, patients with altered gut microbiome resulting from broad-spectrum antibiotics, prolonged use of certain antiepileptics, hyperlactatemia, and elevation of urine 3-hydroxyisovalerate and 3-methylcrotonylglycine in urine organic acid profile may be considered compelling indications to start biotin supplementation. However, serum biotin level is not a sensitive biomarker of biotin deficiency [103].

### 2.7. Vitamin B9

Vitamin B9 (folate) is important for neurodevelopment and is utilized as a cofactor in most metabolic systems in the body. Folate is required to synthesize purines and thymidylate and for the remethylation of homocysteine to methionine. In addition, folate is essential for epigenetic gene regulation through its connection to the methylation cycle and the production of RNA, DNA, monoamine neurotransmitters, nitric oxide, and ATP through its relation to purine production [104].

Deficiencies in folate are well known to cause macrocytic anemia, neuropsychiatric manifestations, and, in the fetus, neural tube defects [16]. Unfortunately, folic acid, the synthetic form of folate used to fortify food, is the oxidized inactive form of folate, which cannot readily enter the folate cycle and may inhibit folate metabolism when excessive. Thus, medical therapies utilize reduced folates, such as leucovorin (folinic acid) and 5-methyltetrahydrofolate (5MTHF), which can readily enter the folate cycle.

ASD is associated with polymorphisms in genes encoding the dihydrofolate reductase, reduced folate carrier, and methylenetetrahydrofolate reductase proteins [105]. Autoantibodies to the folate receptor α, the primary mechanism transporting folate into the brain, are associated with ASD [106] with an estimated prevalence of 71% [14]

Most clinical trials on ASD have used leucovorin, primarily because of the decades of experience of its use in medicine in oncology and rheumatology. A recent meta-analysis found that leucovorin improves neurological symptoms as well as core and associated ASD symptoms, particularly language, in children with ASD [14].

Folate is usually derived from the diet, but many countries fortify food with folic acid, the oxidized inactive form of folate. Thus, reduced folates, which can enter the folate cycle readily, are used in medical treatment. Folate is transported across cell membranes with various transporters, which can be dysfunctional, resulting in disease. Enzymes in the folate cycle convert folate into its various forms [107].

The recommended dietary allowance of dietary folate equivalents ranges from 65–600 mcg, with the maximum upper limit at 1000 mcg. Most folate preparations are oral, but folate can also be intravenous as an intramuscular or subcutaneous injection. In general, folates are well tolerated without known long-term adverse effects [107], although hyperactivity can occur during the initiation phase of leucovorin treatment in ASD [108]. ASD-specific multivitamins range in folate doses from 500 mcg to over 3000 mcg daily. Studies using leucovorin range in dosage from 0.5–2 mg/kg/day, with case reports using 4 mg/kg/day for those with mitochondrial disorders [107].

### 2.8. Vitamin B12

Vitamin B12 (cobalamin) is essential for neurodevelopment and is utilized as a cofactor in most metabolic systems. Adenosylcobalamin is the coenzyme of methylmalonyl-CoA mutase, while methylcobalamin is required for methionine synthase that remethylates homocysteine into methionine. Cobalamin deficiency is known to cause macrocytic anemia, peripheral neuropathy, neuropsychiatric manifestations, and memory impairment [16]. As mentioned above, blood concentrations of homocysteine and methylmalonic acid, the metabolic precursors to reactions, are more sensitive than B12 blood concentrations to diagnose B12 deficiency [109].

Dietary intake of B12 may be lower in children with ASD [9]. Serum B12 is significantly lower in individuals with ASD [10], and one study found that methyl-B12 (mB12) concentration was markedly lower in brain tissue in individuals with ASD [13]. In addition, polymorphisms in the gene encoding cobalamin binding protein (TCN2) are associated with ASD [110], and some individuals with TNC2 mutations have ASD [111]. Perhaps most significantly, B12 is the cofactor for methionine synthase, a critical enzyme in the methylation cycle essential to produce methionine, a precursor of SAMe, the cell’s principal methyl donor [112].

As outlined in a recent systematic review and meta-analysis, 17 studies, including four DBPC studies, have examined the therapeutic use of oral or injectable B12 for ASD, with most using an injectable form of B12 and the great majority of studies using the mB12 form [112]. Studies have examined the therapeutic effect of B12 on transmethylation, transsulfuration, and behavior. A meta-analysis found that subcutaneously injected methyl-B12 improved transsulfuration, and, in general, cobalamin improves a wide variety of core and associated ASD symptoms and medical problems such as sleep and GI problems [112].

Vitamin B12 is available in several forms. Cyanocobalamin is a synthetic form that is not found in nature. Methylcobalamin, hydroxycobalamin, and adenosylcobalamin are naturally occurring forms with better bioavailability than cyanocobalamin [113]. Absorption of cobalamin is sensitive to GI abnormalities as it requires intrinsic factor, a protein secreted in the stomach necessary for cobalamin absorption. Cobalamin is commonly provided orally or injected subcutaneously or intramuscularly; sublingual commercial forms are available, and B12 is not uncommonly compounded into a transdermal gel or intranasal spray. Subcutaneously injected B12 dose ranged from 64.5 to 75 µg/kg/dose given every one to three days, and oral B12 dose ranged from 500–1600 µg daily in clinical studies. Several alternative delivery methods exist for B12 since enteral absorption requires the binding protein intrinsic factor produced in the stomach. As many children with ASD have GI disorders, the oral route is not preferred, although many ASD-specific multivitamins include B12.

### 2.9. Vitamin C

Vitamin C (L-ascorbic acid and its oxidation product dehydroascorbic acid) can be considered the most potent water-soluble antioxidant that scavenges free radicals, moderates oxygen radical production, and recycles other antioxidants, acting as a potent reducing agent. It is also a cofactor for the biosynthesis of neurotransmitters, including serotonin [16], and additionally found to improve mood [114]. As humans are incapable of de novo synthesis of vitamin C, dietary intake strictly determines the body level, and studies have suggested the requirement of supplementing in patients at risk [16]. Neuropsychiatric or developmental disorders such as ASD are considered risk factors for ascorbic acid deficiency owing to their restrictive dietary patterns, as described in several case reports in recent years [115,116,117]. Furthermore, vitamin C levels were found to be higher and lower in 29% and 13% of individuals with ASD, respectively, compared to the controls [27]. Clinical symptoms of vitamin C deficiency (scurvy) have been reported in ASD [29,117], and a DBPC trial in 18 children with ASD reported reduced stereotyped behaviors with vitamin C treatment [118]. In a recent systematic review by Sharp et al. and a case study by Masci et al., all patients with vitamin C deficiency and ASD were found to have an aversion to fruits and vegetables with symptomatic improvement following supplementation with vitamin C [119,120]. The daily recommended dose of vitamin C in patients at risk of deficiency is 500 mg, while 50–100 mg/day is adequate for a healthy individual [16].

### 2.10. Vitamin E

Several isoforms of vitamin E have been identified; α-, β-, γ-, δ- tocopherols and α-, β-, γ-, δ-tocotrienols. Linked to the other antioxidants in the redox-antioxidant cycle (vitamin C, glutathione, glutathione peroxidase, glutathione reductase, etc.), vitamin E inhibits the peroxidation of membrane lipids by scavenging highly reactive peroxyl radicals [121].

Individuals with ASD have significantly lower vitamin E levels than healthy subjects [10,122,123], while the levels correlated with the severity of the social and cognitive impairment measures [124]. Correspondingly, vitamin E has been suggested as a biomarker in predicting the occurrence of ASD [125]. In some studies, the examination of food records revealed that male ASD individuals have a significantly higher percentage of inadequacy in vitamin E [126]. Conversely, a meta-analysis revealed that individuals with ASD exhibit higher vitamin E levels than controls [9]. In a single group assignment study involving patients with verbal apraxia, 97% reported dramatic improvements in several areas, including speech, imitation, coordination, eye contact, behavior, sensory issues, and development of pain sensation following vitamin E combined with polyunsaturated fatty acid supplementation [127]. Further studies on larger sample sizes are warranted to explore the effects of vitamin E on core and co-occurring symptoms in ASD. Deficiency or toxic effects of vitamin E are rare. In ASD, vitamin E may be given at a 200–400 IU daily dose [39].

### 2.11. Vitamin D

Cholecalciferol (vitamin D3) and ergocalciferol (vitamin D2) are animal-derived and plant-derived forms of vitamin D, respectively. The storage form in the body is 25-hydroxy vitamin D (25(OH)D/calcidiol). The active forms of vitamin D are calcitriols, 1,25-dihydroxy vitamin D3, and 1,25-dihydroxy vitamin D2. They are pleiotropic secosteroid hormones that regulate the gene expression of more than 900 genes by binding to the vitamin D receptor that acts as a nuclear transcription factor. The well-known biological functions of calcitriol include regulation of calcium absorption, promoting bone mineralization, modulation of cell proliferation and differentiation, and immune response. Additionally, more recent studies have demonstrated that vitamin D also regulates brain development in early life and contributes to synaptic plasticity, neuroprotection, neural circuitry, and turnover of the dopaminergic system in adults [128].

In two systemic reviews/meta-analyses, vitamin D levels were reported to be lower in children diagnosed with ASD compared to children in the control groups, with significant heterogeneity [129,130]. Meta-regression indicated that latitude was associated with the mean difference, although it can only account for 8% heterogeneity. Interestingly, subgroup analysis stratified by latitude showed that mean vitamin D concentrations of participants with and without ASD from lower-latitude areas showed the largest differences. This suggests that increasing light exposure in children with ASD does not result in the expected increase in vitamin D levels as in the healthy controls. Moreover, numerous case reports describe ASD patients who developed vitamin D deficiency with or without nutritional rickets due to self-imposed dietary restrictions [29]. Self-imposed dietary restrictions may contribute to the inadequate vitamin D intake reported in individuals with ASD [8,9,12,20]. Furthermore, therapeutic dietary restrictions such as a casein-free diet [131] and long-term administration of antiepileptic drugs [132] in some ASD cases may also contribute to vitamin D deficiency.

Meta-analysis of maternal and neonatal vitamin D concentration indicated a trend of lower concentration in individuals with ASD [130]. Another meta-analysis based on the highest vs. the lowest category of prenatal 25(OH)D levels demonstrated that higher prenatal concentrations were associated with a lower risk of ASD, autism-related traits, and attention deficit hyperactivity disorder (ADHD), a commonly associated comorbid condition [133]. Therefore, low vitamin D status in utero, postnatally, and in early childhood may be implicated in neurodevelopmental and cognitive outcomes in infants [134].

According to a meta-analysis of data from three studies, vitamin D supplementation was beneficial for hyperactivity but not for core ASD symptoms or irritability [135]. Correspondingly, in a patient with ASD, core symptoms did not improve following the correction of vitamin D deficiency [136]. Conversely, in some reported cases, core symptoms improved significantly after vitamin D supplementation [137,138]. Furthermore, a case series reported that core symptoms of three patients with ASD fluctuated in severity with changes in serum 25(OH)D levels, indicating that core symptoms in some vitamin D deficient-ASD subjects might be responsive to vitamin D therapy [139]. Accordingly, these contrasting observations suggest the presence of two phenotypes depending on how core symptoms respond to treatment; vitamin D-responsive vs. vitamin D non-responsive. Vitamin D levels in ASD subjects have been reported to correlate positively with intelligence [140]. Further research is needed to explore whether intelligence improves after correcting vitamin D deficiency. The therapeutic effect of vitamin D may be related to increasing brain estrogen levels [141], enhancing the tryptophan-serotonin-melatonin pathway [142], modulating mTOR signaling [143], decreasing neuroinflammation, increasing glutathione levels, and upregulating neurotrophins [144].

The presence of vitamin D deficiency appears to be a compelling indication to start supplementation. Patients on a casein-free diet should be investigated for vitamin D deficiency [131]. Vitamin D can be administered through oral or intramuscular routes, and toxicity is rare [145]. Symptoms of toxicity are mediated by high calcium levels. The drug dosage is estimated according to the age and body weight of the individual. Clinical trials indicate that 300 IU/kg/day (Max. 6000 IU/day) of vitamin D3 is well tolerated [146]. Individuals with ASD may have blunted serum vitamin D response to supplementation owing to various factors. Therefore serum 25(OH)D levels should be regularly measured to assess the response to therapy [147].

### 2.12. Vitamin A (Retinol)

Vitamin A (Retinol) is a prohormone converted to two active metabolites: retinoic acid and retinal. It is essential for normal growth and development, vision, immunity, reproduction, and cellular differentiation in mucous membranes. Vitamin A exerts its effects through its direct derivative, retinoic acid, which augments multiple gene modulation [148,149]. Retinoid metabolism involves different biochemical forms of the vitamin, including retinyl esters, retinol, retinal, retinoic acid, and oxidized and their conjugated metabolites. Being a lipid-soluble vitamin, it is transported in serum mainly bound to retinol-binding protein, a negative acute-phase protein [148]. Therefore, assaying vitamin A levels should be avoided during infection or inflammation.

Many studies have demonstrated that vitamin A correlates with cognitive function, spatial learning, and memory which are some of the parameters of underperformance in children with ASD compared to neurotypical children [150,151,152,153]. ASD children have a higher risk of nutritional deficiencies due to abnormal food habits, which are highly prevalent in ASD. Many studies have demonstrated reduced vitamin A intake and serum concentrations below the reference range and lower than neurotypical children [12,154,155,156,157,158]. One study identified vitamin A as the most seriously deficient nutrient among children with ASD [154]. These studies have further stated that vitamin A levels inversely correlate with Aberrant Behavior Checklist (ABC), Social Responsiveness Scale (SRS), and Childhood Autism Rating Scale (CARS) scores, suggesting the possible correlation of vitamin A deficiency with ASD symptoms [12,154]. Correlating to the pathophysiology of ASD in relation to vitamin A deficiency, researchers have found that retinoid acid receptors that act as ligands for gene activation show a low expression level in children with ASD compared to neurotypical controls [149].

Substantial evidence has emerged supporting the possible role of vitamin A acting as a modulator for the tryptophan hydroxylase-1 gene in regulating serotonin levels. Serotonin contributes to neuropathological symptoms in ASD in higher concentrations [149,159,160].

Supplementary interventional studies support the significant improvement of core symptoms with vitamin A supplementation, specifically emotional response, sensory perception, and communication [149]. It was also noted to have contributed to a reduction in serum serotonin levels, which were found to be consistently elevated in ASD [155].

In inadequate vitamin A consumption, liver stores are sufficient for about six months [16]. Several oral and intramuscular vitamin A preparations are available for supplementation and pharmacological use. Because of the high bioavailability of vitamin A oral preparations, the provision of large doses at longer intervals is recommended as it is well absorbed, stored in the liver, and mobilized on demand over an extended period [149,161]. The recommended dose of vitamin A in infants 6–11 months is 100,000 IU, while it is 200,000 IU in children 12–59 months, to be given at 6-month intervals [161].

The recommended dose is well tolerated in children, although occasional transient adverse effects such as headache, nausea, vomiting, and diarrhea, which typically last for about 24 h, have been reported [161]. Much more serious, acute toxicity develops when serum vitamin A levels reach >60,000 IU in children [162]. Acute intoxication results in increased intracranial pressure, nausea, headache, and joint and bone pain [3,16]. Chronic toxicity can result from daily ingestion of vitamin A >25,000 IU for more than six years or >100,000 IU for more than six months [163]. Hepatotoxicity can result from the intake of vitamin A >14,000 μg/d for extended periods [16].

## 3. Minerals

### 3.1. Zinc

In the human body, zinc (Zn^2+^) functions as a structural, catalytic, and regulatory co-factor for thousands of proteins. Over 300 enzymes use zinc, and over 2500 transcription factors have a zinc finger domain to bind to DNA [164,165]. Pre- and perinatal zinc deficiencies have repeatedly been related to ASD in epidemiological research [11,166,167]. Studies examining the effects of zinc deficiency reveal that zinc has a special function in the nervous, GI, endocrine, and immune systems [168,169,170]. For instance, in the GI system, zinc regulates the microbiota composition and influences the tightness of the intestinal barrier and immunological signaling [171,172]. Because zinc has a direct function in the central nervous system, notably at synapses, as well as through effects on the GI and immune system, low zinc status during brain development may contribute mechanistically to the development of ASD [173,174,175,176,177].

Zinc supplementation might be used as a preventative measure and, possibly, a therapeutic approach to lessen the symptoms and comorbidities associated with ASD [178]. Notably, zinc supplementation was able to reverse the behavioral abnormalities in various rodent models of ASD [179,180,181,182,183].

Zinc supplements are classified as “inorganic zinc” (zinc sulfate, zinc oxide, zinc acetate, etc.), “organic zinc” (zinc-polysaccharide, zinc amino acid conjugates (ZnAAs), etc.), and “biologically organic zinc” (zinc-enriched yeast, seaweed zinc extracts, etc.). Due to their mode of absorption, organic zinc supplements may be more beneficial for those with ASD [178].

Vitamin A and zinc have an interdependent relationship. Vitamin A deficiency can impair zinc absorption. This is thought to result from the reduced synthesis of vitamin A-dependent zinc-binding protein in ileal mucosa. On the other hand, zinc plays a regulatory role in vitamin A transport via the synthesis of retinol-binding protein, a cofactor role in the oxidative conversion of retinol to retinaldehyde [184]. Therefore, taking vitamins A and D with zinc has been identified as a strategy to augment zinc absorption [185]. Nevertheless, the vitamin A, D, and zinc combination has not been studied in ASD.

### 3.2. Magnesium

Magnesium is a cofactor for over 600 enzymes, an allosteric regulator of many enzymes, and is proposed as a regulator of the mammalian target of rapamycin (mTOR) complex. As a regulator of N-methyl-D-aspartate (NMDA) receptor excitability in the brain, magnesium plays a role in glutamate-mediated excitatory synaptic transmission, neuronal plasticity, and excitotoxicity hence a vital role in developmental plasticity, learning, and memory [186]. Hypomagnesemia is associated with migraine, depression, schizophrenia, bipolar disorder, neuroses, addiction, neurodegenerative diseases, and epilepsy [186,187]. Magnesium is essential for many ATP-dependent enzymes, including many enzymes of glycolysis [186] and S-adenosylmethionine synthetase [188], which is required for SAMe synthesis.

A meta-analysis found non-statistically significant lower blood and erythrocyte magnesium levels in ASD, but significantly decreased magnesium levels in the serum and hair (after removal of an outlier ASD individuals), as compared with controls [11].

Transdermal administration through Epsom salt baths or magnesium sulfate cream has been used in children with ASD to stimulate detoxification, reduce inflammation, promote healthy circulation, and normalize sleep patterns [189]. It may also be given as trace amounts spread throughout the day to prevent diarrhea. This may compensate for the sulfate deficiency observed in children with ASD [190]. Nevertheless, the positive benefits appear to be mediated by magnesium and the sulfate components. Epsom salt baths were part of a complex regimen with multiple supplements and dietary interventions in a study of ASD [191]. There is limited evidence that magnesium alone is useful in treating ASD. Magnesium-vitamin B6 co-therapy is discussed in a subsequent section.

As enzymes dependent on thiamine also require magnesium as an additional cofactor [30], taking magnesium with thiamine is also recommended and may help absorption and utilization [192].

### 3.3. Vitamin B6-Magnesium Combination

The main enzymes involved in the metabolism of vitamin B6, including PDXK, ALPL, and pyridoxal phosphate phosphatase, require magnesium as a cofactor. Furthermore, the transmethylation pathway and transsulfuration pathway of sulfur amino acid metabolism require magnesium and PLP as cofactors, respectively. Magnesium bioavailability is increased by the concomitant intake of vitamin B6 [193]. Pyridoxal phosphate but not pyridoxal, appears to form a complex with magnesium and hence may enhance the transport or accumulation of magnesium in cells [194].

PDXK regulation by thiamine is of particular interest. Thiamine inhibits PDXK, an enzyme that activates vitamin B6 (a non-cofactor role of thiamine). This reaction seems to inhibit vitamin B6 metabolism in the presence of magnesium, acting differently from zinc [195]. This may explain why magnesium may reduce the adverse effects of high doses of vitamin B6, such as irritability, sound sensitivity, and enuresis [196]. This association has only started to be examined. However, it would be fortuitous to learn if thiamine could protect against vitamin B6 toxicity.

Earlier DBPC trials did not produce significant effects overall but showed significant benefits for IQ in a subgroup of children with the pervasive developmental disorder. Hence no recommendation was advanced in a Cochrane review [197]. In subsequent studies, overall improvement in core ASD symptoms has been reported with vitamin B6-magnesium therapy [198,199].

### 3.4. Molybdenum

In humans, molybdoenzymes such as aldehyde oxidase, xanthine oxidoreductase, and sulfite oxidase require a molybdenum cofactor for enzymatic function. Interestingly, the activity of xanthine oxidase is proportional to the amount of molybdenum in the body [16].

Several case reports provide evidence for ASD phenotype in patients with molybdenum cofactor deficiency [200,201]. A subgroup of ASD exhibits significantly high urine sulfite levels, lower plasma levels of sulfate, and decreased sulfation capacity compared to neurotypicals, suggesting there could be a problem with sulfite oxidase activity [190]. Conversely, a meta-analysis reported significantly higher hair molybdenum levels in patients with ASD compared with control subjects [11]. Urinary sulfite and sulfate levels have improved following supplementation with molybdenum [190]. Furthermore, in a randomized DBPC treatment with a vitamin/mineral formula containing molybdenum, whole-blood molybdenum levels significantly increased, potentially explaining the changes in sulfate levels [21]. This suggests a functional defect in molybdenum cofactor biosynthesis in the absence of molybdenum deficiency.

A controlled study in four healthy young men found that molybdenum intakes up to 1490 mg/day (almost 1.5 mg/day) elicited no serious adverse effects when molybdenum was administered for 24 days. In individuals living in areas with extremely high soil contents, adverse effects such as aching joints, gout-like symptoms, hyperuricosuria, and elevated blood molybdenum have been reported with intakes of 10–15 mg/day [16].

### 3.5. Selenium

Selenium (Se) is required to synthesize the amino acid selenocysteine, an essential component of selenoproteins such as glutathione peroxidases which participate in antioxidant activity in the extra- and intracellular compartments. Selenoproteins are also involved in the control of cell proliferation and apoptosis [16]. Interestingly there are sex differences in selenium metabolism. Males have faster kinetics and are more sensitive to the toxic effect of excessive selenium intake. This has implications for designing clinical trials on the effect of selenium in ASD. The role of selenoenzymes in relation to the pathogenesis of ASD has received much attention [202].

A meta-analysis reported that hair or erythrocyte selenium concentrations of individuals with ASD were not significantly different from controls [11]. However, a recent meta-analysis concluded that selenium levels in the hair of children with ASD were higher than those of healthy children [167]. On the other hand, ASD patients showed 18% lower glutathione peroxidase levels in erythrocytes [105]. Interestingly, a study revealed an unexpected negative correlation between changes in selenium levels following a ketogenic diet and behavioral scores, suggesting that selenium may relate to improving ASD symptoms with the ketogenic diet. Correspondingly, supplementation with selenium inhibits ferroptosis, attenuates ASD-like behaviors, and improves oxidative stress and inflammation through modulation of related gene expression in the BTBR ASD mouse model [203,204]. Clinical trials in ASD should be highly advocated.

Selenoenzymes, such as thioredoxin reductase and glutathione reductase, engage in converting ubiquinone to ubiquinol. [205]. Therefore, selenium may work synergistically with CoQ_10_ in therapy.

### 3.6. Copper

Most of the body’s copper is present as oxidized cupric (Cu^2+^). It is an essential catalytic cofactor for oxidation-reduction reactions involving copper-containing enzymes (e.g., Cu/Zn-superoxide dismutase). Copper enzymes are integral to energy production (cytochrome C oxidase), iron metabolism (ceruloplasmin), neurotransmission (dopamine metabolism), and regulation of peptide hormones. In addition, copper is essential for cholesterol, thyroid hormone and glucose metabolism, and aspects of immune function [16].

According to meta-analyses, copper concentrations were significantly increased in children with ASD relative to neurotypical subjects [10,167]. In contrast, copper was observed to be reduced in hair [167]. The altered copper level is often discussed in terms of the zinc/copper ratio and may indicate a zinc deficiency, copper toxicity, and a resultant dysfunctional metallothionein system [166]. Adams et al. reported that more than 10% of individuals with ASD had copper levels in whole blood or red blood cells above the normal reference range [27], and the same study group did not include copper in the vitamin/mineral supplement used for their randomized DBPC trial [21].

### 3.7. Iron

Iron, the most abundant trace element in the human body, is present in ferrous (Fe^2+^) and ferric (Fe^3+^) states. Iron is a functional component of heme. Heme participates in oxygen binding and transport (hemoglobin, myoglobin), oxidation-reduction reactions (catalases, peroxidases), cellular respiration, and electron transport (cytochromes). Proteins containing non-heme iron also play a significant role in DNA synthesis, cell proliferation, cell differentiation, gene regulation, drug metabolism, and steroid synthesis. Iron is also essential for both innate and adaptive immunity [16].

The redox cycling of ferrous and ferric iron, in the presence of H_2_O_2_ can contribute to the production of hydroxyl radicals through the Fenton reaction, that in turn, induces lipid peroxidation. This process is identified as a possible culprit in the pathogenesis of ASD [27,206]. Meta-analysis of comparisons between ASD and control groups showed no difference in blood iron levels but lower iron levels in the hair of patients with ASD [11]. A scoping review reported that three studies found statistical evidence for an association between iron deficiency and ASD severity, while three studies did not. Further contradictory results were reported in studies investigating the prevalence of iron deficiency and iron deficiency anemia [207]. A study demonstrated that hemoglobin levels were significantly lower in ASD patients with intellectual disabilities and severe ASD [208]. No randomized controlled study has demonstrated the efficacy of iron supplementation in improving core symptoms of ASD or associated intellectual disability.

Iron deficiency is the most common nutritional deficiency worldwide. The deficiency leads to impaired physical and cognitive functions [16]. Therefore, the prevalence of iron deficiency anemia in ASD may be as common as in the general population or even higher due to selective eating habits and GI comorbidities frequently observed in ASD. Taken together, iron supplementation in ASD should be considered in individuals with iron deficiency anemia and/or low ferritin levels.

## 4. Other Cofactors

### 4.1. Coenzyme Q_10_

Coenzyme Q_10_ (CoQ_10_) is the only lipid-soluble antioxidant endogenously synthesized that exists in two forms, the oxidized form ubiquinone and the reduced form ubiquinol. It is an obligatory inner mitochondrial membrane co-factor that plays a crucial role in connecting Complex I and II with Complex III in the electron transport chain [209], activating uncoupling proteins, regulating permeability, and modulation of the physicochemical properties of mitochondrial membranes [210], and recycling of the other antioxidants such as vitamin C and E and thereby protecting membrane phospholipids against peroxidation [211]. Importantly it can become depleted in the context of oxidative stress, especially in the context of mitochondrial dysfunction. Furthermore, CoQ_10_ also affects the expression of several genes related to cell signaling, intermediary metabolism, and embryonic development and exhibits a potential epigenetic role [212].

CoQ_10_ is synthesized from tyrosine and acetyl-CoA through reactions that require folate, cobalamin, riboflavin, niacin, and pyridoxine with the last synthesis step requiring the methyl donor SAMe; hence its deficiency can result from the deficiency of any of these vitamins and cofactors which is pertinent in ASD [213].

Interestingly, children with ASD have been shown to have depressed ubiquinone [214], suggesting that supplementation with ubiquinol may be therapeutic for at least a subgroup of children with ASD. CoQ_10_ is a basic component of the “mitochondrial cocktail” [215] used to support individuals with mitochondrial dysfunction, as has been identified in many children with ASD [214]. However, only two clinical studies have examined the effect of CoQ_10_ alone. In a large (*n* = 90) DBPC study, a low dose of ubiquinol (30–60 mg) improved GI problems and sleep disorders as well as markers of oxidative stress in children with ASD [216]. In a small (*n* = 24) open-label study, ubiquinol (100 mg) improved communication, playing, sleeping, and food rejection in a minority of study participants if the CoQ_10_ plasma level was above 2.5 umol/L [217]. Several other studies, which have used CoQ_10_ along with other supplements that support mitochondrial and redox metabolism, have shown improvements in both core and associations ASD symptoms. These include DBPC [191], cross-over open-label [22], and retrospective studies [218]. One study suggested that the combination of melatonin and CoQ_10_ improves fatty acid metabolism in an animal model of ASD [219]. A recent meta-analysis and systematic review suggest CoQ_10_ may have a role in improving sleep for children with ASD [220].

In general, ubiquinol demonstrates greater bioavailability than ubiquinone [221] and twice the blood levels [222] compared to ubiquinone. CoQ_10_ is given orally. Ubiquinol is usually given at 100–400 mg per day in two divided doses; ubiquinone is given at 2 to 10 times higher doses of ubiquinol.

### 4.2. Alpha-Lipoic Acid

Alpha-lipoic acid is an essential prosthetic group of several mitochondrial enzymes, such as pyruvate dehydrogenase complex, 2-oxoglutarate dehydrogenase complex, glycine cleavage system H protein, and branched-chain alpha-keto acid dehydrogenase complex. It is not considered a vitamin because humans can generate lipoic acid in mitochondria using lipoyltransferase 1 (LIPT1), lipoyltransferase 2 (LIPT2), and lipoic acid synthase (LIAS) [223]. Synthesis begins with octanoic acid (best known as caprylic acid) or cysteine. Intestinal microbes, diet, or supplements may also furnish lipoic acid. Interestingly, alpha-lipoic acid behaves quite similarly to biotin. Both will form a moving arm that is attached via a lysine in a protein, formed with an interaction with an iron/sulfur center, and share the SMVT transporter to cross the cell membranes. But alpha-lipoic acid may also cross membranes via the monocarboxylic transporter (MCT) [224].

Lipoic acid is a significant antioxidant, switching between oxidized and reduced states via the action of enzymes like dihydrolipoamide dehydrogenase, changing between alpha-lipoic acid or dihydrolipoic acid [223]. An elaborate system keeps the reduced form regenerated, calling upon the antioxidant network of Vitamin E, Ubiquinol, Ascorbic acid, and Glutathione, as well as the cofactor NADPH [225]. It scavenges reactive oxygen species, regenerates or induces other antioxidants, or induces the Nrf2/ARE pathway. Lipoic acid can chelate redox-active metals and is an activator of many proteins, including PKC, Erk1/2, p38 MapK, PI3K, and Akt.

Lipoic acid has varied roles in the immune system, including inhibiting NFκB, keeping inflammatory cells from crossing the blood-brain barrier, and inhibiting LFA-2, ICAM1, VLA-4, VCAM-1, and MMP-9. It decreases levels of both TNF-alpha and IFN-gamma and neutralizes reactive oxygen species and NO [226]. It also activates the insulin receptor and activates AMPK, which is the critical regulator of AMP-activated protein kinase. It also can inactivate PTEN, PP2A, and PTB1b [227]. In ASD, it has tended to be used mostly for its antioxidant and chelation properties.

Lipoic acid is quickly absorbed and has a short half-life in plasma that may argue for frequent and divided dosing. Food may block absorption, so lipoic acid should be taken 30 min before or 2 h after eating. It is extremely safe at high doses, with a lethal dose of 2 g per kg. Doses may range from as low as 200 mg/day to 2 g/day, but 1200 mg/day has been studied in many clinical trials [228].

### 4.3. Tetrahydrobiopterin

Tetrahydrobiopterin is an essential cofactor for several critical metabolic pathways, including synthesizing monoamine neurotransmitters, such as serotonin and dopamine, that are focal points in ASD. It is also critical for degrading aromatic amino acids such as phenylalanine and tryptophan [229]. Tetrahydrobiopterin is a naturally occurring pteridine but cannot be derived from the diet in quantities necessary to support metabolism. Thus, the production of pterins, including biopterin, sepiapterin, neopterin, and tetrahydrobiopterin, is necessary for metabolism. It is synthesized de novo from guanosine-5′-triphosphate, a purine nucleotide derived from the folate cycle [230]. Tetrahydrobiopterin is available as an oral formulation known as sapropterin dihydrochloride, with an orphan drug designation and is costly at this time.

In one study, tetrahydrobiopterin concentrations were depressed in the cerebrospinal fluid of children with ASD, especially those less than six years of age [229]. Investigation into the therapeutic use of tetrahydrobiopterin in ASD started over three decades ago with open-label studies, which motivated two blinded controlled studies [229,231]. One biomarker study suggested that the therapeutic effect of tetrahydrobiopterin in ASD may be primarily due to the stabilization of nitric oxide production [232].

## 5. Discussion

**Figure 1:**
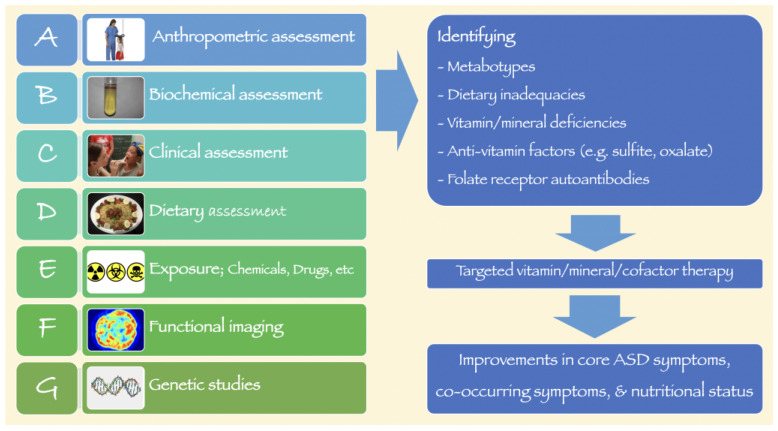
The multimodal and integrated approach (A to G approach) to determine the best vitamin/mineral/cofactor therapy in autism spectrum disorder (ASD).

Children with ASD carry a high risk of vitamin and cofactor deficiency for many reasons. Selective eating behaviors could be a barrier to meeting some nutritional requirements. Accumulation of toxic metabolites such as sulfite may cosume vitamins. Long-term administration of antiepileptic drugs may induce vitamin deficiencies. Furthermore, the presence of antibodies against vitamin transporters at the blood-brain barrier leads to cerebral vitamin deficiencies. Specific metabolic phenotypes such as increased oxidative stress, mitochondrial dysfunction, or reduced methylation capacity may indicate an increased nutritional demand. Therefore, a thorough assessment through a multimodal and integrated approach is needed to determine the best vitamin/s, mineral/s, and cofactor/s that would bring about the desirable therapeutic effects (Figure 1).

Clinical features of a vitamin/mineral deficiency, biochemical findings suggestive of absolute or relative nutritional deficiency, and self-imposed or therapeutic dietary restrictions are compelling indications for considering vitamin and other cofactor supplementation in ASD. Moreover, certain inborn errors of metabolism (e.g., biotinidase deficiency) associated with ASD may respond to cofactor supplementation by correcting the homeostasis and enhancing the residual enzyme activity.

Besides reducing core ASD symptoms, nutritional supplementation may also improve co-occurring symptoms and optimize the nutritional status. Supplementation is also advisable to prevent the deficiencies induced by ketogenic and restriction diets. Doses higher than that typically required for the cofactor functions may be necessary to bring about the therapeutic effects. The dose and specific forms of vitamins should be wisely used based on bioavailability and potential toxicity. Nutritional supplements should be meaningfully combined to correct specific metabolic abnormalities such as oxidative stress, neuroinflammation, mitochondrial dysfunction, etc. Using combinations of vitamins may have numerous advantages over monotherapy, such as increasing bioavailability, synergistic action, and alleviation of adverse effects of high doses used in ASD. Unfortunately, only a few studies have explored the impact of meaningful micronutrient combinations in ASD. Therefore, it is necessary to design clinical trials to evaluate the efficacy of the combinations.

## 6. Conclusions

The current review intends to highlight a significant pitfall in clinical trials. ASD is highly heterogeneous in terms of the metabotype. Therefore, vitamin and cofactor therapies should be personalized in line with the endophenotype of the child. Conducting clinical trials on the general ASD population without subgrouping based on the metabotype can yield statistically insignificant effects on the “typical” comparison of outcomes between “two arms,” leading to underestimation of the efficacy of therapy when it may have been remarkably effective in a certain subgroup had the study conducted with appropriate subgrouping. This could explain the inconsistent findings of some trials in ASD. Therefore, investigators should commence a study with subgroup analysis to identify the exophenotype or endophenotype that respond to the therapy of interest. Further, retrospective metabolic investigations can be performed to recognize individuals who respond to specific therapies to identify the endophenotypes leading to better and more informed future studies.

## Data Availability

Not applicable.
